# Lamin B1 curtails early human papillomavirus infection by safeguarding nuclear compartmentalization and autophagic capacity

**DOI:** 10.1007/s00018-024-05194-3

**Published:** 2024-03-14

**Authors:** Freya Molenberghs, Marlies Verschuuren, Lauran Vandeweyer, Sarah Peeters, Johannes J. Bogers, Claudina Perez Novo, Wim Vanden Berghe, Hans De Reu, Nathalie Cools, Mario Schelhaas, Winnok H. De Vos

**Affiliations:** 1 Laboratory of Cell Biology and Histology, Department of Veterinary Sciences and Health Sciences, University of Antwerp, Universiteitsplein 1, 2610 Antwerp, Belgium; 2 Cell Death Signaling Lab, Integrated Personalized and Precision Oncology Network (IPPON), Department of Biomedical Sciences, University of Antwerp, Antwerp, Belgium; 3 Laboratory of Experimental Hematology, Faculty Medicine and Health Sciences, University of Antwerp, Antwerp, Belgium; 4 Institute of Cellular Virology, University of Münster, Münster, Germany

**Keywords:** Human papilloma pseudovirus, Nuclear lamina, Lamin B1, Infection kinetics, Mitotic window, Nuclear rupture, Autophagy

## Abstract

Human papillomavirus (HPV) infection is a primary cause of cervical and head-and-neck cancers. The HPV genome enters the nucleus during mitosis when the nuclear envelope disassembles. Given that lamins maintain nuclear integrity during interphase, we asked to what extent their loss would affect early HPV infection. To address this question, we infected human cervical cancer cells and keratinocytes lacking the major lamins with a HPV16 pseudovirus (HP-PsV) encoding an EGFP reporter. We found that a sustained reduction or complete loss of lamin B1 significantly increased HP-PsV infection rate. A corresponding greater nuclear HP-PsV load in *LMNB1* knockout cells was directly related to their prolonged mitotic window and extensive nuclear rupture propensity. Despite the increased HP-PsV presence, EGFP transcript levels remained virtually unchanged, indicating an additional defect in protein turnover. Further investigation revealed that *LMNB1* knockout led to a substantial decrease in autophagic capacity, possibly linked to the persistent activation of cGAS by cytoplasmic chromatin exposure. Thus, the attrition of lamin B1 increases nuclear perviousness and attenuates autophagic capacity, creating an environment conducive to unrestrained accumulation of HPV capsids. Our identification of lower lamin B1 levels and nuclear BAF foci in the basal epithelial layer of several human cervix samples suggests that this pathway may contribute to an increased individual susceptibility to HPV infection.

**Graphical abstract:**

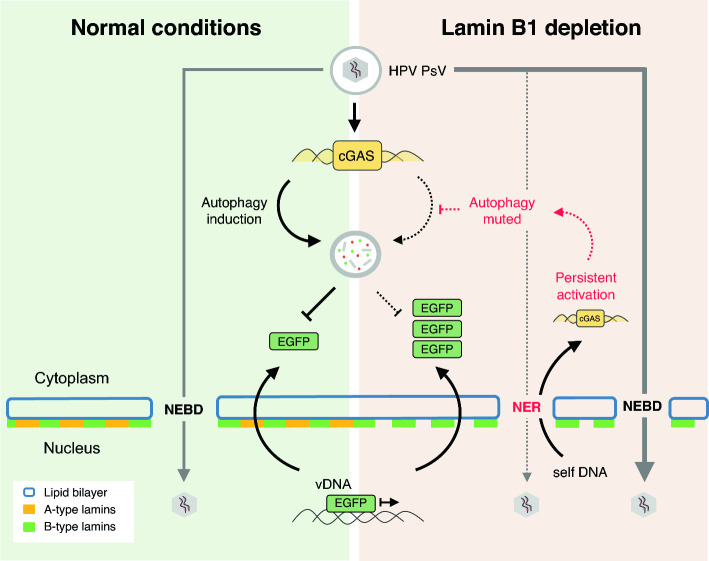

**Supplementary Information:**

The online version contains supplementary material available at 10.1007/s00018-024-05194-3.

## Introduction

Human papillomaviruses (HPV) are small non-enveloped DNA viruses. Several HPV types cause anogenital cancers and a growing number of tumors of the head and neck. HPV16 is the most prevalent and best-studied high-risk type and it is the primary etiological agent of cervical cancer [1]. HPV particles have a spherical, icosahedral structure with a diameter of 50–55 nm. Their 8-kb circular double-stranded viral DNA (vDNA) genome is surrounded by a capsid built up of 72 homo-pentameric capsomers of the major structural protein L1 and a variable number (12–72 copies) of the minor structural protein L2 [2]. HPV particles infect the basal keratinocytes of the cutaneous or mucosal epithelia and their viral replication depends on the differentiation of these cells into mature keratinocytes [3].

Like all DNA viruses, HPV exploits the host cell’s DNA processing machinery for its own replication [4]. However, to reach the nucleus, viruses need to pass a sequence of barriers, such as the plasma membrane, the limiting membrane of an intracellular organelle after endocytosis, the cytosol and, eventually, the nuclear envelope (NE) [5, 6]. To cross the NE barrier, HPV awaits mitotic NE breakdown (NEBD). This view is supported by a large-scale RNA interference (RNAi) screen unveiling mitotic regulators such as AURKB, ANAPC and INCEBP as major drivers for HPV infection in HeLa cells [7] and experiments showing that chemical inhibition of cell cycle progression abrogated infection [7, 8]. Throughout early infection, the incoming HPV virus resides and traffics in a vesicular compartment [9]. Upon its arrival in the nucleus, the viral protein L2 and the vDNA will graft promyelocytic leukemia protein (PML) nuclear bodies (NBs). PML protects vDNA from the innate immune recognition and degradation at the early stages of the HPV life cycle, and fosters transcription and replication [10].

At its intranuclear face, the NE is delineated by a dense meshwork of intermediate filament proteins, called lamins. The A-type lamins (lamins A and C) are encoded by the *LMNA* gene [11], whereas the B-type (lamins B1 and B2) are encoded by the *LMNB1* and *LMNB2* gene, respectively. Lamins organize the genome and regulate its expression [12], but they also confer mechanical resilience [13, 14] and define the viscoelastic properties of the nucleus [15]. The loss of nuclear lamins has been shown to provoke changes in the nuclear compartmentalization [13] such as deregulation of the Ran gradient [16, 17]. It is conceivable that such events predispose cells to enhanced viral nuclear entry during interphase. Conversely, several viruses have been shown to rely on lamins or their interactors for efficient nuclear entry [18, 19]. For example, during nuclear entry, HIV viral Vpr protein triggers local disassembly of the nuclear lamina, resulting in localized herniations in the NE that burst occasionally [20], while parvoviruses (hyper)phosphorylate nuclear lamins to trigger local lamin depolymerization [19]. Herpesvirus HCMV viral proteins pUL50 and pUL53 can induce structural modifications of the nuclear lamina in order to complete nuclear egress [21].

We set out to shed a light on the contribution of lamins to HPV nuclear entry. To this end, we selectively depleted individual lamins in cervix carcinoma cells lines, after which we challenged them with a HPV16 pseudovirus (HP-PsV) expressing a fluorescent reporter. We found that reduction or complete loss of *LMNB1* enhances HP-PsV infection, but without significantly increasing the number of HP-PsV transcripts. By systematically dissecting the mechanism in *LMNB1ko* cells, we uncovered an autophagic defect that leads to unrestrained HP-PsV protein buildup.

## Materials and methods

### Cell culture

HeLa cells (ATCC) and HaCaT cells (DKFZ, German Cancer Research Center, Heidelberg, Germany) were cultured in DMEM high glucose with L-glutamine and pyruvate (Gibco, 41966-029), supplemented with 10% fetal bovine serum (Gibco, 10270-106) and 1% penicillin/streptomycin (Gibco, 15140-122), according to standard procedures. For HPV16 PsV production, 293TT cells (National Cancer Institute, Rockville, USA) were cultured in DMEM high glucose, L-glutamine and pyruvate (Gibco, 41966-029) supplemented with 400 µg/ml Hygromycin B (Merck-Millipore, 400052) and 10% fetal bovine serum (FBS). Proliferative capacity was monitored by cell counting with every passage and cultures were tested regularly for the most common mycoplasma infections using a PCR test kit (Bio-connect, PK-CA91-1024).

### HPV16 PsV production

HPV16 PsV (HP-PsV) were produced by co-transfecting 293TT cells with p16SheLL and pClneo-eGFP or p8RwB plasmids (Addgene plasmid #37320, plasmid #46949 and plasmid #48733, all a kind gift of Dr. John Schiller) [22–24]. The protocol used has been previously described [25]. For PsV labeling experiments, the growth medium of the cells was supplemented with 20 µM 5-ethylnyl-2′-deoxyuridine (EdU) five hours after the transfection. Viral titers were expressed in terms of viral genome equivalents (VGE) and were determined based on the protocol of Biryukov [26].

### siRNA gene silencing

siRNA mediated knockdown (kd) of *LMNA* (LMNAkd) (Dharmacon®, D-001050-1-20), *LMNB1* (LMNB1kd) (Dharmacon®, M-005270-01-0005), *LMNB2* (LMNB2kd) (Dharmacon®, M-005290-00-0005) and *PML* (PMLkd) (Dharmacon®, M-006547-01-0005) was performed using Lipofectamine® RNAiMAX (Thermo fisher scientific, 13,778,075) according to manufacturer’s instructions. Cells transfected with non-targeting siRNA (Thermo fisher scientific, 12935300) were used as control cells (CTRLkd). Three subsequent transfection steps were executed every 48 h, to guarantee sustained kd [27]. The first kd was executed (time point 0 h) 24 h after cell seeding. Quantification of the kd efficiency was performed at different time points (i.e., 48 h, 96 h, 144 h and 192 h) by immunofluorescence staining (IF) and western blot (WB).

### CRISPR/Cas9 genome editing

Stable knockout (ko) HeLa cell lines for *LMNA* (LMNAko), *LMNB1* (LMNB1ko) and *LMNB2* (LMNB2ko) were produced with CRISPR/Cas9-mediated genome editing. gRNA and Cas9 encoding plasmids were delivered to the cells using Lipofectamine 2000 (Life Technologies, 11,668,027). The plasmids were constructed starting from pSpCas9(BB)-2A-Puro (PX459) (from Feng Zhang, Addgene #48139). The gRNA sequence, targeting the first exon of the gene, were: 5′- CCTTCGCATCACCGAGTCTGAAG -3′ for *LMNA*, 5′- CCGTGCCCATCCGCGGCGGCACG -3′ for *LMNB1* and 5′- CCGGTCGATGTAGTGCGCCAGG -3′ for *LMNB2*. They were designed with the CRISPR oligo design tool (Feng Zhang), based on the protocol of Ran et al. [28]. Control cells (CTRLko) were treated identically as the ko cells but using a construct containing no gRNA. Cells were selected 24 h after transfection using 3 µg/ml puromycin. Next, single cells were selected using a FACSAria II (Becton Dickinson). After clonal expansion, three to four different colonies of ko cells were pooled to account for clonal heterogeneity. Validation of ko was done by IF and WB.

### Transfection

For live cell imaging, cells were transfected with an expression plasmid encoding NLS-EYFP (generous gift from Dr. J. Goedhart, University of Amsterdam, the Netherlands) and cGAS-RFP (generous gift from Dr. J. Lammerding, Cornell University, USA) using Lipofectamine 2000 (Life Technologies, 11668027) according to the manufacturer’s instructions.

### Compound treatment

The proteasome inhibitor MG132 (Tocris Bioscience, 1748) was used as a positive control in the proteasomal degradation assay at a concentration of 2 µM for 24 h. The autophagy inducer Rapamycin was used as a positive control in the autophagy activity assay at a concentration of 2 µM in combination with Chloroquine, a lysosomal activity inhibitor, at a concentration of 10 µM for 24 h (both part of the CYTO-ID® autophagy detection kit (Enzo, ENZ-51031-K200)).

### HPV16 PsV infection studies

Cells were seeded 24 h before infection at 5 × 10^4^ cells/well in 12-well glass bottom plates (Cellvis, P12-1.5H-N), 2 × 10^4^ cells/compartment in 4-well glass-bottom dishes (CELLview™, Greiner) or 5 × 10^3^ cells/well in 96-well plates (iBL America, 220.230.043), and infected with HPV16 EGFP PsV, EdU-labelled HPV16 EGFP PsV or HPV16 RwB PsV at a MOI of 100–400 VGE/cell. The cells were incubated with HP-PsV for 2 h on a shaker at 37 °C. Afterwards the medium of the cells was removed, and new medium was added. For live cell imaging, HP-PsV was added just prior to imaging and medium was left untouched throughout the imaging procedure.

### Immunofluorescence staining (IF)

For HPV infection studies, cells were fixed with 4% paraformaldehyde (freshly made) for 25 min followed by 3 × wash step with PBS (Life technologies, 14190-169). After permeabilization in 0.3% Triton X-100 (Sigma, X100-500 ml), cells were washed again 3 × with PBS before staining with DAPI (1 µg/ml) for 15 min. Plates were maintained in PBS-NaN_3_ at 4 °C pending microscopic imaging. For IF, cells were blocked in 50% FBS after the permeabilization for 30 min and primary antibodies were added for one h. Following primary antibodies were used: mouse anti-lamin A/C (Santa Cruz Biotechnology, sc-376248, 1/100), rabbit anti-lamin B1 (Abcam, ab16048, 1/250), rabbit anti-lamin B2 (Abcam ab151735, 1/500), rabbit anti-PML (Santa Cruz, sc5621, 1/250), mouse anti-H3K9me2,3 (Cell Signaling Technology, #5327, 1/100), rabbit anti-H3K9ac (Cell Signaling Technology, #9649, 1/400), mouse anti-IRF3 (Abcam, ab68481, 1/100) and rabbit anti-p65 (Abcam, ab7970, 5 µg/ml). After 3 × 5 min wash step with PBS, the secondary antibodies; donkey anti-mouse CY3 (Jackson, 715-165-151, 1/600) and donkey anti-rabbit CY5 (Jackson, 711-175-152, 1/600), were added for 30 min. Afterwards cells were washed again 3 × 5 min with PBS and stained with DAPI similar as above. For transcription factor localization experiments additional HCS CellMask™ staining (Life Technologies, H32721, 1/5000) was applied, to allow distinguishing nuclear from cellular signal. To visualize EdU-labeled pseudogenomes, we made use of the Click-iT EdU Alexa Fluor™ 555 imaging kit (Invitrogen, C10338). Cells were incubated for 30 min at room temperature with the Click-iT reagent after blocking. Anonymized archival paraffin embedded human cervix samples, were microtome sectioned onto SuperFrost slides, deparaffinized (xylene) and rehydrated. After antigen retrieval (citrate buffer), they were subjected to an immunostaining for lamin A/C, lamin B1 and/or BAF (Abcam, ab129184, 1/500), counterstained with DAPI and mounted with Citifluor.

### Western blot (WB)

Cells were grown in 6-well plates (Greiner Bio-One, 657102) or 12-well plates (Thermo Scientific, 150628) and lysed using M-PER® Mammalian Protein Extraction Reagent (Thermo scientific, 78503). Protein concentration was measured with the Pierce™ BCA Protein Assay Kit (Thermo scientific, 23227). Cell lysates were mixed with 25% NuPage LDS sample buffer (ThermoFisher, NP0007) and 5% dithiothreitol (DDT, ThermoFisher NP0009) and heated for 10 min on 70 °C. Samples (5 µg) were loaded onto NuPAGE™ Novex 4–12% Bis–Tris Protein Gels (ThermoFisher, NP0322PK2), with MOPS running buffer (Thermo Scientific, J00047). PageRuler P-prestained Protein Ladder was used as marker (ThermoFisher, PI26616). Next, proteins were transferred to BioTrace PVDF membranes (Pall Corporation, 66542) using a transfer mixture of NuPAGE transfer buffer (ThermoFisher), NuPAGE antioxidant (ThermoFisher) and methanol. Afterwards the membranes were blocked in blocking buffer (5% ECL (Sigma GERPN418) in Tris Buffered Saline with 0.2% Tween 20 (TBST)), and subsequently incubated with primary antibodies, diluted in blocking buffer. The following primary antibodies were used: mouse anti-lamin A/C (Santa Cruz Biotechnology, sc-376248, 1/100), rabbit anti-lamin B1 (Abcam, ab16048, 1/1000), rabbit anti-lamin B2 (Abcam ab151735, 1/1000), mouse anti-H3K9me2,3 (Cell Signaling Technology, #5327, 1/1000), rabbit anti-H3K9ac (Cell Signaling Technology, #9649, 1/1000) and anti-cGAS (Cell Signaling Technology, 15102S, 1/1000). Rabbit anti-Nucleolin (Novus Biologicals, NB600-241, 1/4000) and anti-GAPDH (GeneTex, GT239, 1/10000) were used as a reference protein. Horse radish peroxidase (HRP)-conjugated goat anti-mouse (Sigma-Aldrich A4416, 1/5000) and HRP-conjugated goat anti-rabbit (Sigma-Aldrich A6154, 1/5000) were used as secondary antibodies. Proteins were detected by chemiluminescence with Immobilon western chemiluminescent HRP substrate (Millipore, WBKLS0100) using a western blot Imager (Bio-Rad, ChemiDocTM XRS +). Quantification was done with Fiji image processing freeware [29] by measuring the intensity of each band in a rectangular selection of fixed size and the intensity of each marker band was expressed relative to that of the corresponding reference protein in the same lane.

### Proteasomal activity measurement

Proteasomal activity was measured using the Proteasome 20S activity assay kit (Abcam, ab112154), according to manufacturer’s instructions. The assay was extended by adding Hoechst staining to allow for normalization for cell density. Cells were seeded at 8.000 cells per well in 96-well µClear plates (Greiner Bio-One, 655090). At 24 h, medium was discarded and 100 µl of fresh medium or medium containing chemical treatment was added to the cells. At 45 h, 100 µl proteasome assay solution, supplemented with Hoechst, was added, and incubated for 3 h at 37 °C and 5% CO_2_. Fluorescence was measured using a Fluoroskan™ plate reader (Thermo Fisher) with excitation/emission set at 490/525 nm for GFP and 340/480 nm for Hoechst.

### Autophagic capacity measurement

Autophagic vacuoles in untreated and Rapamycin/Chloroquine-treated CTRLko and LMNB1ko cells were quantified using the CYTO-ID® autophagy detection kit (Enzo, ENZ-51031-K200), according to manufacturer’s instructions. Cells were seeded at 4000 cells per well in 96-well µClear plates. At 24 h, cells were infected with HPV PsV RwB, 24 h later, chemical treatment was initiated. At 72 h, cells were washed with 1 × assay buffer after which the detection solution containing CYTO-ID green detection reagent together with Hoechst 33342 was added and incubated for 30 min in the incubator. Afterwards, cells were washed again twice with 1 × assay buffer and fixed with 4% paraformaldehyde for 25 min, followed by a final wash step.

### Flow cytometry

After 24 h of infection, cells were trypsinized and resuspended in PBS with 1% EDTA and 1% FBS for fluorescence-activated cell sorting (FACS). For the collection of RNA of EGFP-positive HPV PsV infected CTRLko and LMNB1ko cells, cells were also selected based on their EGFP signal with the use of BD FACSAria II, using an exciting 488 nm laser and detecting emission with a 530/30 laser. Control cells were included to set the correct gates and a gating strategy was created to remove doublets and debris.

### RT-qPCR

RNA was extracted from EGFP-positive and EGFP-negative HP-PsV infected (+ HP-PsV EGFP + /EGFP-) but also from pooled HPV PsV infected CTRLko and LMNB1ko cells using RNeasy Micro Kit (Qiagen, 74004), which was followed by cDNA synthesis using iScript™ cDNA Synthesis Kit (Bio-Rad, 1708891). Afterwards RT-qPCR was executed using EGFP primers: forward: 5′-AGAAGAACGGCATCAAGGTG-3′, reverse: 5′-GAACTCCAGCAGGACCATGT-3′.

### Kinase activity profiling

A Serine/Threonine kinase PamChip® (Pamgene International BV, Hertogenbosch, The Netherlands) was used to profile activity of Serine/Threonine kinases in PsV-infected vs. non-infected Hela cells. Each pamchip contains four identical peptide arrays with each array containing 144 peptide sequences. Phosphorylation of these peptides by kinases present in the cell lysates is detected by means of fluorescently labelled anti-phospho antibodies in a PamStation12, allowing the inference of putative kinases from the phospho-signature [30].

Proteins were extracted from HP-PsV-infected HeLa cells (at 48 h post infection (PI)) and non-infected HeLa cells. Media of these cells was removed, and cells were washed with ice cold PBS. Afterwards, ice cold PBS was added again to the cells and cells were harvested with the use of a cell scraper. Cell suspension was centrifuged for 5 min at 250 g at 4 °C. The cell pellet was dissolved in ice cold PBS and an equal number of cells (over the different conditions) were again centrifuged. The cell pellet was resuspended in a lysis buffer containing M-PER® Mammalian Protein Extraction Reagent, Halt™ Phosphatase Inhibitor Cocktail (Thermo Fisher Scientific, 78420) and Halt™ Protease Inhibitor Cocktail, EDTA-Free (100x) (Thermo Fisher Scientific, 87785) (both 100 × diluted).

Protein concentrations were quantified using the Pierce™ BCA Protein Assay Kit to assure equal protein loading onto the PamChip®. Three technical replicates were executed in the same run but on different chips for each condition. Data analysis was executed in the BioNavigator software as described before [30] and in R.

### Microscopy

For automated widefield microscopy, cells were seeded in 12-well glass bottom plates or 96-well plates. Imaging was performed on a fully automated Nikon Ti Eclipse inverted widefield fluorescence microscope, equipped with a Perfect Focus System and LED-based illumination source. For the HPV infection study, at least three wells were used as technical replicates for a given condition. Per well, 32 regions were monitored, using a 10×/0.30 Plan Fluor dry lens or 24 regions were monitored using a 20×/0.75 Plan Fluor dry lens. 405 nm, 488 nm, 561 nm and 650 nm LED illumination was used for excitation of DAPI, EGFP, CY3 and CellMask or CY5, respectively. Detection was done through a quadruple dichroic using 395/25, 470/24, 555/25 and 640/30 band pass filter, respectively, with a DS-Qi2 CMOS camera.

For live cell imaging, cells were seeded in 4-well glass-bottom dishes or 96-well plates 24 h before imaging. Two h prior to imaging, cells were stained with Silicon-Red Hoechst (SiR-DNA, Spirochrome, sc007) after which medium was replenished. Time-lapse imaging was performed on a Perkin Elmer Ultraview Vox dual spinning disk confocal microscope, mounted on a Nikon Ti body, equipped with a Perfect Focus System II and a microscope incubator equilibrated at 37 °C and 5% CO_2_. Recordings were made every 10 min, using a 20×/0.75 Plan Achromat dry lens. 640 nm and 488 nm diode lasers were used for the excitation of SiR-DNA and EGFP, respectively. Detection was done on a Hamamatsu C9100-50 camera using Volocity (Perkin Elmer) software, by using 525/50–705/90 bandpass emission filters. Per well, 10 regions were monitored, meaning that 40 different regions were imaged every 10 min for 48 h. The same system was used for acquiring images of immunostained human biopsy sections.

Time-lapse imaging was also performed on a Nikon CSU-W1-01 SoRa spinning disk confocal microscope, mounted on a Nikon Ti Eclipse body, equipped with a Perfect Focus System and a microscope incubator equilibrated at 37 °C and 5% CO_2_. Recordings were made every 3 min, using a 20 × /0.75 Plan Apo dry lens. Per well 6 regions were monitored, meaning that 48 different regions were imaged every 3 min for 16 h. 488 nm (excitation of NLS-EYFP) and 640 nm (excitation of SiR-DNA) diode lasers and 520/35 nm and 685/40 nm bandpass filters were used. Images were acquired with a Prime 95B camera.

The same confocal microscope was used in superresolution-by-optical-pixel-reassignment (SoRa) mode for the detection of AF555-labeled EdU or cGAS-RFP using a 561 nm diode laser and 617/35 nm bandpass emission filter. DAPI was excited with a 405 nm diode laser and detected through a 447/60 nm emission filter. In this setting, images were acquired using a 20 × /0.75 Plan Apo dry lens or 40 × /0.95 Plan Apo dry lens with a Kinetix sCMOS camera.

### Image analysis

Image analysis was performed in FIJI image analysis freeware [29]. An in-house developed image analysis pipeline (https://github.com/DeVosLab/CellBlocks) was used to detect nuclei in fixed assays and live cell imaging in the nuclear counterstain channel (DAPI or SiR-DNA) using a trained convolutional neural network as implemented in the StarDist plugin [31–33]. Using the nuclear regions as seeds, cytoplasm was detected using a user-defined threshold for the CellMask channel. Both morphological and intensity measurements were extracted from detected nuclear and cytoplasm regions. A Laplacian operator of fixed scale was used to selectively enhance spots in the PML channel, CYTO-ID channel (autophagy assay) or EdU channel (labelled HP-PsV), prior to their detection with a user-defined fixed threshold.

For tracking cells through time, we made use of the TrackMate plugin (version 7) [34]. Various particle detection algorithms are integrated in this version, including Stardist [31]. However, to tweak the nuclear detection we pre-processed the images by exploiting StarDist-mediated nucleus detection included in our analysis pipeline [31] and conversion of the detected nuclear ROI into indexed spots of 2 µm radius (https://github.com/VerschuurenM/StarTrack) [25]. Within TrackMate, we limited the linking distance during tracking to 20 µm, allowed gap closing of max. 20 µm over 2 frames and allowed track splitting within a distance of 20 µm.

### Data analysis

Data analysis and representation was done in RStudio [35]. All plots were made using the ggplot2 package. Infected cells were identified by setting a user-defined intensity threshold on the raw EGFP intensity. Based on this, the ratio of infected cells to the total number of cells was calculated. To compare infection ratios and average nuclear EGFP intensities, linear mixed effects models with independent experimental replicate as a random factor and technical replicate (well for fixed assays) as nested factor were used (*lme4::lmer*). These models were combined with the Tukey method to control the family-wise error during multiple comparisons. Analogues models were used to statistically compare nuclear PML occupancy, the number of autophagic vacuoles in the cell and the chromatin condensation status. Since normality and homoscedasticity could not be assumed, a non-parametric Kruskal Wallis test was used to compare differences in mitotic window and viral load in LMNB1ko cells when individual nuclei were used as data points. The symbols in the figures indicate statistically significance (p < 0.05). The exact p-values can be found in Supplementary Table 1.

For live cell imaging, HP-PsV infection kinetics were quantified based on a logistic function (Eq. 1) [36] that was fit on both the infection ratio (defined as the percentage of EGFP positive cells in the field of view) as well as the nuclear EGFP intensity (EGFP positive population) using the Levenberg–Marquardt algorithm implemented in the *forestmangr:nls_table* function. The beginning of the log-linear phase was calculated based on the maximum of the second derivative of the fitted function [37]. Other used parameters were the maximum infection ratio (defined as *a* in Eq. 1) and the slope (defined as *b* in Eq. 1).

1 $$y=\frac{a}{1+{e}^{-b(t-c)}}+d$$

## Results

### Lamin B1 depletion increases HP-PsV infection rate

To determine the role of nuclear lamins during HP-PsV infection, we selectively depleted nuclear lamins in HeLa human cervix carcinoma cells. A sustained knockdown (kd) of *LMNA*, *LMNB1* and *LMNB2* was obtained using repetitive siRNA-mediated gene silencing, while stable *LMNA*, *LMNB1* and *LMNB2* knockout (ko) cells were produced using targeted CRISPR/Cas9-mediated genome editing (Suppl. Fig. S1a). We compared lamin-depleted cells with mock-treated controls (CTRLkd/ko), which underwent identical operational procedures, using a scrambled (non-sense) nucleotide sequence (kd) or no guide (ko). Effective depletion was validated at the protein level using quantitative immunofluorescence and qualitative western blotting (Suppl. Fig. S1b–e). Next to the virtual absence of the targeted lamins, canonical lamin-specific morphological aberrations of the cell nuclei were observed. Depletion of A-type lamins (LMNAkd, LMNAko) resulted in wildly dysmorphic nuclei with local loss of B-type lamins, while the depletion of lamin B1 (LMNB1kd, LMNB1ko) was associated with characteristic nuclear bleb formation, most evident in the lamin A/C staining. Depletion of lamin B2 (LMNB2kd, LMNB2ko) did not result in a clear divergent phenotype (Suppl. Fig. S1b). Quantitative IF validation of nuclear parameters confirmed these nuclear aberrations. Circularity, for example, was significantly increased in LMNB1ko cells and decreased in LMNAko cells. LMNB1ko cells also displayed a significant increase in lamin A/C intensity (Suppl. Fig. S1c).

**Fig. 1:**
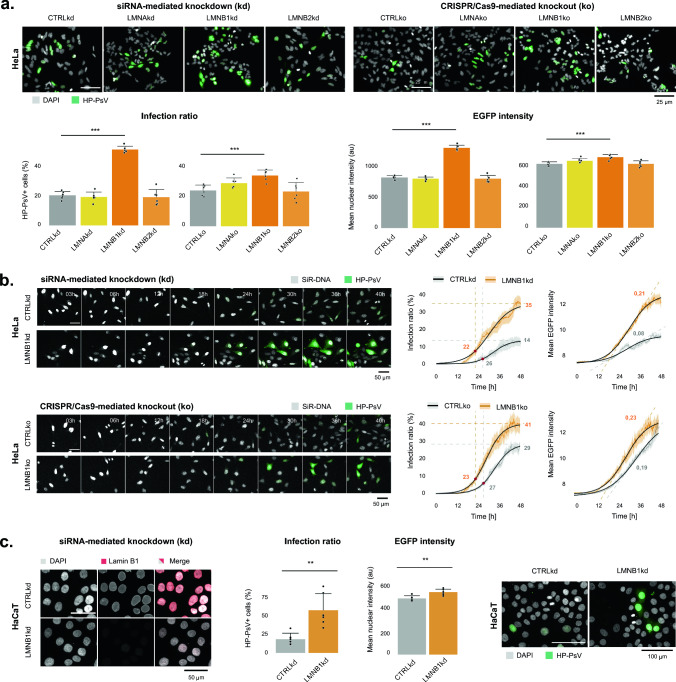
Increased HP-PsV infection ratio upon depletion of lamin B1. **a** LMNB1kd cells display significantly increased infection ratio (fraction of EGFP-positive cells) and mean EGFP intensity compared to CTRLkd cells 48 h post-infection (left panel). The same increase, albeit less extreme, is present in LMNB1ko cells (right panel) (n_bio_ = 2, n_tech_ = 3, p < 0.05, linear mixed effects model); **b** Montages of live cell imaging and corresponding quantification of HP-PsV infection kinetics in HeLa cells. The infection ratio defined as the percentage of EGFP-positive cells in the field of view, as well as the nuclear EGFP intensity of the EGFP positive cell population monitored over time and fitted with a logistic function. The start of the log-linear phase of infection occurs earlier in *LMNB1* depleted cells compared to CTRL cells. In addition, nuclear EGFP intensity in EGFP-positive cells increases faster in *LMNB1* depleted cells compared to CTRL cells (n_bio_ = 2, n_tech_ = 10); **c)** Sustained siRNA-mediated knockdown of *LMNB1* in HaCaT cells leads to a drastic reduction in lamin B1 levels and significantly increases HP-PsV infection ratio and EGFP intensity

To determine their susceptibility to infection, lamin-depleted cells were infected with HP-PsV encoding an EGFP reporter. 48 h after infection the fraction of EGFP-positive cells – a proxy for infection rate—was quantified using automated microscopy. In the panel of kd cells, no differences were observed with CTRLkd cells, except for the LMNB1kd cells which displayed a very significant > two-fold increase in the fraction of EGFP-positive cells (Fig. 1a). A similar, albeit less extreme (~ 1.5-fold), increase was observed in LMNB1ko cells (Fig. 1a). Live cell imaging revealed an earlier onset of HP-PsV infection in LMNB1ko cells as compared to CTRLko cells, resulting in a higher infection ratio 48 post-infection (Fig. 1b). Also, the absolute EGFP intensity of individual LMNB1ko cells increased faster and more after HP-PsV infection than in CTRLko cells (Fig. 1b). While HPV has a natural tropism for keratinocytes, we have previously shown that HeLa is a much more susceptible and robust model for infection studies [25]. Yet, to ascertain that the observed increase was not specific to the cell line at hand, we infected HaCaT human keratinocytes with HP-PsV. Also in these cells, a significant increase of both the infection ratio and EGFP intensity was observed after siRNA-mediated downregulation of *LMNB1* (Fig. 1c). When plotting the EGFP intensity at the single cell level, we found a global increase irrespective of the nuclear size, suggesting there is no specific subpopulation more susceptible to infection (Suppl. Fig. S2). Thus, we conclude that depletion of lamin B1 causes a general increase in HP-PsV infection rate.

### Lamin B1-depleted cells have an extended nuclear access window

**Fig. 2:**
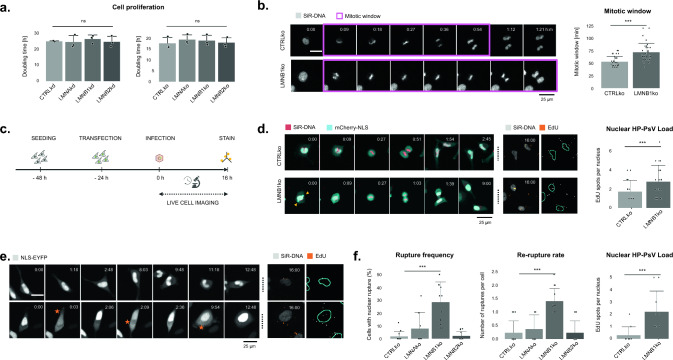
Enhanced nuclear access due to prolonged mitotic window and nuclear ruptures in lamin B1 depleted cells. **a** Cell proliferation, expressed as population doubling time, does not significantly differ between lamin-depleted cell lines (n_bio_ = 3) (error bars = SD); **b** The mitotic window, measured from prophase until late telophase, is significantly longer (72 ± 18 min) in LMNB1ko cells than in CTRLko cells (53 ± 10 min) (n_cell_ = 35, Kruskal–Wallis test, using pairwise post-hoc tests with Benjamini-Hochberg correction); **c** Schematic workflow. After seeding, CTRLko and LMNB1ko cells are transfected with NLS-EYFP and infected with EdU-labeled HP-PsV. Live cell imaging (for 16 h) is immediately followed by fixation and staining to visualize EdU positive foci; **d** Quantification of EdU spots in cells that divided once during live cell imaging reveals a higher number in LMNB1ko compared to CTRLko daughter cells (i.e., 2.8 ± 1.7 vs. 1.8 ± 1.2 EdU nuclear spots, respectively) (n_cell_ = 20, Kruskal–Wallis test, using Pairwise post-hoc tests with Benjamini-Hochberg correction, p < 0,05); **e**, **f** LMNB1ko cells display an increased nuclear envelope rupture (NER) and re-rupture rate during interphase, as evident from the transient loss of nuclear NLS-EYFP signal and concomitant increase in cytoplasmic signal (orange stars indicate NER) (n_bio_ = 2, n_tech_ = 6) (error bars = SD), linear mixed effects model). While hardly any EdU spots are seen in CTRLko cells that have not undergone division during the 16 h imaging window (i.e., 0.3 ± 0.7), a significantly higher number of EdU spots is present in LMNB1ko cells (2.2 ± 1.7) (n_cell_ = 10, Kruskal–Wallis test, using Pairwise post-hoc tests with Benjamini-Hochberg correction). The contrast of the images has been globally adapted for visualization purposes

Since mitosis is crucial for HPV nuclear entry [7, 8, 25], a change in cell cycle kinetics may alter the infection probability. However, none of the lamin depleted cells—whether established by sustained knockdown or stable knockout—displayed an altered population doubling time (Fig. 2a). Since the latter only provides a rough readout and does not inform on shifts in the duration of individual cell cycle phases, we diverted to live cell imaging to measure the length of the mitotic window (from prophase till late telophase). We made use of SiR-DNA to visualize the chromosomes and an NLS-EYFP reporter to visualize the loss and restoration of nuclear compartmentalization during mitosis (Fig. 2c). While the mitotic window of CTRLko cells was 53 ± 10 min, LMNB1ko displayed a significantly extended mitotic window of 72 ± 18 min (Fig. 2b). Using EdU-labeled HP-PsV, we found that LMNB1ko cells with prolonged mitosis had a significantly higher HP-PsV load in their nuclei compared to CTRLko cells (i.e., 2.8 ± 1.7 EdU vs. 1.8 ± 1.2 EdU nuclear spots, respectively) (Fig. 2d). During live cell imaging, it also became apparent that LMNB1ko cells suffer from frequent nuclear envelope rupture (NER) events, which lead to temporary loss of compartmentalization during interphase (Fig. 2e). While these events are short-lived (minute scale), they were much more frequent in LMNB1ko cells than in the other cell lines and they were found to occur more repetitively in the same cell (Fig. 2f). We therefore asked whether this could contribute to the higher HP-PsV load as well. Using EdU-labeled HP-PsV, we discovered that LMNB1ko cells which had undergone repetitive NER but no mitosis, indeed displayed a significantly higher number of EdU spots (*i.e.,* 2.2 ± 1.7 for LMNB1ko cells, while 0.3 ± 0.7 for CTRLko) (Fig. 2f). Therefore, we conclude that LMNB1ko cells have a higher HP-PsV load as they offer more access to the nuclear compartment due to a prolonged mitotic window and repetitive NER events.

### HP-PsV DNA transcription does not scale with infection rate in LMNB1ko cells.

A higher HP-PsV load and increased EGFP signal would suggest more transcription of HP-PsV DNA in LMNB1ko cells. To verify this, we infected CTRLko and LMNB1ko cells with HP-PsV and after 24 h, we performed RNA extraction and qPCR for EGFP transcripts (Suppl. Fig. S3a). Surprisingly, despite a trend towards increase, no significant difference was detected in relative fold expression levels between the cell lines, even when only considering flow-sorted EGFP-positive cells (Suppl. Fig. S3b). This implies that LMNB1ko cells produce a similar number of EGFP transcripts, despite the higher HP-PsV load.

### LMNB1ko cells display a shift in epigenetic state.

**Fig. 3:**
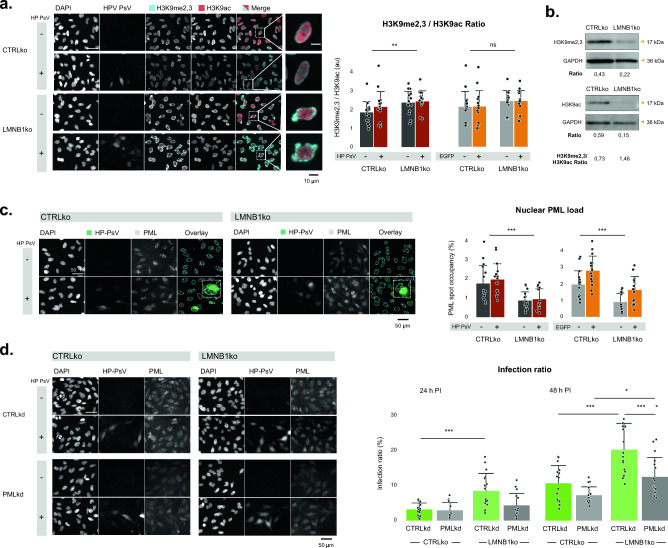
More repressed chromatin and decreased nuclear PML body content in lamin B1 depleted cells. **a** IF of non-infected and HP-PsV infected CTRLko and LMNB1ko cells for histone markers H3K9me2,3 and H3K9ac reveals peripheral accumulation of methylation marker anti-H3K9me2,3 in LMNB1 depleted cells, especially around blebs (insets). The nuclear H3K9me2,3/H3K9ac ratio shows significant differences between CTRLko and LMNB1ko cells, but irrespective of the infection status (nbio = 3, ntech = 5, linear mixed effects model); **b** WB of CTRLko and LMNB1ko cells for histone markers anti-H3K9me2,3 and H3K9ac shows an increased H3K9me2,3/H3K9ac ratio in LMNB1ko cells, despite loading difference;** c** LMNB1ko cells show significantly lower basal levels of nuclear PML foci (expressed as spot occupancy) compared to CTRLko cells. Representative images of CTRLko and LMNB1ko cells, with and without HP-PsV (24 h PI), stained for PML are shown left. The overlay shows EGFP signal (green), the nuclear outlines (in cyan) and segmented PML foci (spots) (white) (yellow arrows indicate EGFP-positive cells with larger PML content, the contrast in the PML channel has been globally adapted for visualization purposes). In both cell lines, EGFP-positive (HP-PsV infected) cells display a significant increase in PML spot occupancy compared to EGFP-negative cells 24 h PI, (n_bio_ = 3, n_tech_ = 5, linear mixed effects model); **d** Silencing *PML* in CTRLko and LMNB1ko cells results in the absence of PML spots as visualized with IF and results in lower HP-PSV infection ratio 24 h PI and especially 48 h PI in CTRLko, and even more clearly in LMNB1ko cells (n_bio_ = 3, n_tech_ = 5, linear mixed effects model)

Given the unaltered mRNA levels, we wondered whether LMNB1ko cells would display a change in epigenetic state that contributes to a less favorable environment for HP-PsV transcription. To this end, we determined the ratio of repressed vs. active chromatin using quantitative immunofluorescence and western blotting for the histone markers H3K9me2,3 and H3K9ac, respectively. The H3K9me2,3/H3K9ac ratio was elevated in LMNB1ko cells, which suggests these cells have relatively more repressed chromatin (Fig. 3a,b). When scrutinizing the spatial distribution, we noticed a conspicuous increase of the H3K9me2,3 signal at the nuclear periphery, particularly inside blebs (Suppl. Fig. S4). We also quantified the H3K9me2,3/H3K9ac ratio after HP-PsV infection, but found no significant change, which indicates that the infection *per se* does not alter the global epigenetic state.

### LMNB1ko cells have a lower basal number of nuclear PML foci

Since the transcription and replication of HPV vDNA is promoted by PML [10, 38, 39], we analyzed the nuclear PML body content in HP-PsV-infected cells. We found that the PML spot occupancy, the relative fraction of the nuclear area covered by PML foci, at 24 h of infection was significantly increased in EGFP-positive compared to EGFP-negative cells (Fig. 3c). Conversely, siRNA-mediated depletion of PML drastically blunted the HP-PsV infection rate (Fig. 3d), underscoring its contribution to HP-PsV transcription. Interestingly, LMNB1ko cells displayed a significantly lower basal PML spot occupancy compared to CTRLko cells (Fig. 3c). While the PML spot occupancy also increased with HP-PsV infection, it did not reach that of infected CTRLko cells. These results suggest that LMNB1ko cells offer a less hospitable environment for HP-PsV transcription, which could explain the observed discrepancy between HP-PsV load and EGFP transcript levels.

### *LMNB1* ko reduces autophagic capacity.

**Fig. 4:**
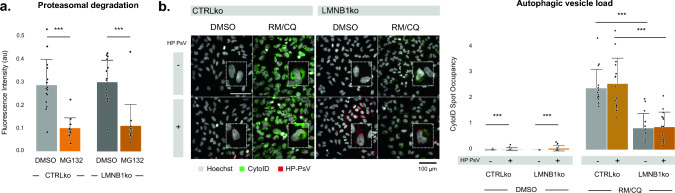
Autophagic capacity is blunted in lamin B1 depleted cells. **a** No differences in proteasomal degradation capacity between CTRLko and LMNB1ko cells, when using proteasome inhibitor MG132 as positive control (n_bio_ = 3, n_tech_ = 5, linear mixed effects model); **b** Fluorescent images of cells stained with CytoID show strong enrichment of autophagic vacuoles after treatment with a combination of Rapamycin (RM) (i.e., inducer of autophagy) and Chloroquine (CQ) (*i.e.,* inhibitor of lysosomal degradation) in CTRLko cells, but much less prominent in LMNB1ko cells. This significant difference in autophagic capacity is also confirmed in the quantification of the cellular CytoID spot occupancy (n_bio_ = 3, n_tech_ = 5, linear mixed effects model)

Despite the higher HP-PsV load, LMNB1ko cells did not produce significantly more transcripts. This implied that the observed higher EGFP intensity in LMNB1ko cells is rather due to a change in protein turnover. We therefore assessed the major protein degradation mechanisms, namely proteasome activity and autophagic capacity in LMNB1ko and CTRLko cells. While no differences were detected in proteasomal activity (Fig. 4a), a significant difference in autophagic capacity was measured (Fig. 4b). The basal number of autophagic vesicles was low in both cell lines, and only modestly (yet significantly) increased after HP-PsV infection in LMNB1ko cells. More strikingly, the maximum autophagic capacity, as induced by combined rapamycin and chloroquine treatment, was significantly lower in LMNB1ko cells (Fig. 4b). A similar decrease was observed in HaCaT cells after sustained knockdown of *LMNB1* (Suppl. Fig. S5). Thus, the blunted autophagic capacity may contribute to the higher EGFP intensity levels observed in LMNB1ko cells.

### cGAS is amply mobilized to the NE of lamin B1 depleted cells

**Fig. 5:**
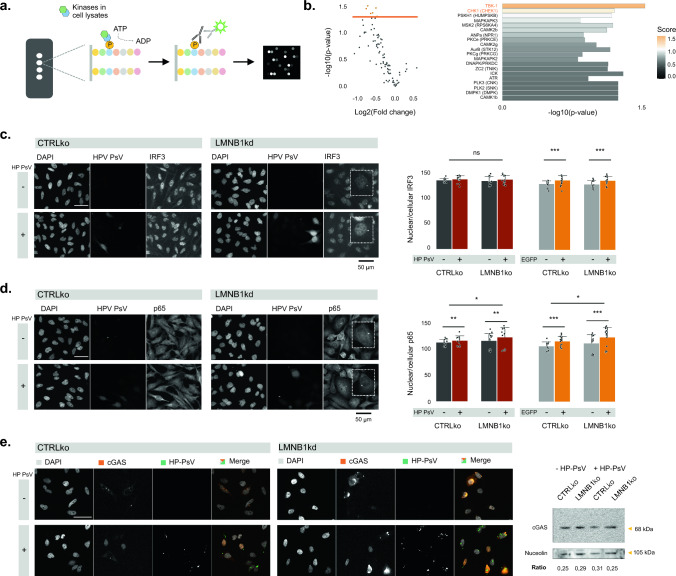
TBK-1 activation in HP-PsV infected cells. **a** Principle of PamGene technology, showing a chip consisting of four arrays with each 144 different peptides. When these peptides become phosphorylated by kinases present in the cell lysate sample, they are bound by fluorescent antibodies resulting in a quantifiable signal intensity; **b** Volcano plot of the peptides showing the log2 fold change on the x-axis, and the -log10(p-value) on the y-axis. In orange peptides—with a p-value < 0.05 were indicated. Upstream kinase analysis based on the total peptide dataset where we show here the top 20 kinases found. TBK-1 was the only kinase with a significant (represented by the normalized kinase statistics) and specific (represented by the specificity score) response in HP-PsV infected (vs. non-infected) HeLa cells **c** EGFP-positive cells display a significantly higher nucleus-to-cytoplasm ratio of IRF3 than EGFP-negative cells, but there is no significant difference between CTRLko and LMNB1ko cells (n_bio_ = 3, n_tech_ = 5, linear mixed effects model); IRF3 localizes to a perinuclear spot solely in LMNB1ko cells (inset); **d** Quantitative IF of TBK-1 downstream target p65 reveals a significantly increased nucleus-to-cytoplasm ratio in EGFP-positive cells compared to EGFP-negative cells as well as between CTRLko and LMNB1ko cells (n_bio_ = 3, n_tech_ = 5, linear mixed effects model); Alike IRF3, p65 also localizes to a perinuclear spot specifically in LMNB1ko cells (inset); **e** Expression of cGAS-RFP, reveals only few cGAS foci in the cytoplasm of CTRLko cells, but strong enrichment at the nuclear periphery, especially in blebs of LMNB1ko cells. HP-PsV infection results in a pan-nuclear signal of cGAS-RFP in both cells. Endogenous cGAS levels detected by western blot do not markedly differ upon HP-PsV infection, nor between CTRLko and LMNB1ko cells

Shotgun serine/threonine kinase activity profiling revealed that TBK-1 is the principal kinase that responds in a significant (represented by the normalized kinase statistic) and specific (represented by the specificity score) manner to HP-PsV infection in HeLa cells (Fig. 5a, b). Since TBK-1 becomes activated upon recognition of vDNA and regulates autophagy [40, 41], we asked whether its downstream signaling would become affected in LMNB1ko cells. To this end, we determined the nuclear translocation of its major transcription factor targets IRF3 and RelA/p65 using quantitative IF. The nucleus-to-cytoplasm ratio of both IRF3 and p65 was significantly higher in EGFP-positive cells compared to EGFP-negative cells (Fig. 5c, d). However, apart from the enrichment of both markers in a conspicuous (but currently undefined) perinuclear spot in LMNB1ko cells, no significant difference was observed in the nucleus-to-cytoplasm ratio with CTRLko cells (Fig. 5c, d; insets). This implies that TBK-1 signaling is still intact in these cells positioning the autophagy defect upstream of this transducer. The initial activation of TBK-1 relies on the detection of dsDNA by the cytosolic DNA sensor cyclic GMP-AMP synthetase (cGAS). Western blot revealed no significant increase in the cGAS levels upon HP-PsV infection nor did it show any clear differences between CTRLko and LMNB1ko cells (Fig. 5e). However, when expressing cGAS-RFP in both cell types, we noticed a marked difference in its localization. Under basal conditions, cGAS signal was limited to sparse cytoplasmic foci, while HP-PsV infected cells showed significantly more cells with pan-nuclear signal. In HP-PsV-infected or non-infected LMNB1ko cells, cGAS was strongly enriched at the nuclear periphery, especially at blebs, consistent with the cytoplasmic exposure of nuclear DNA upon NER (Fig. 5e). Thus, while TBK1 signaling remains functional, cGAS is continuously activated by host DNA in LMNB1ko cells.

### The basal layer of human cervix epithelium recapitulates a low lamin B1 state.

**Fig. 6:**
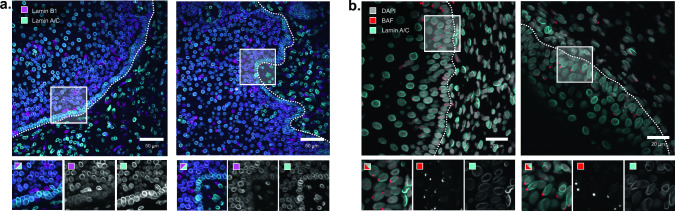
Human cervix samples display lower lamin B1 levels and BAF foci in the basal layer. **a** Representative images of immunostained paraffin sections of a 25-year old human cervix sample stained for lamin A/C and lamin B1 and revealing low, diffuse lamin B1 signal in the basal layer of the stratified epithelium: **b** Representative images of cervix sections from a 23-year old (left) and a 30-year old (right) individual, revealing BAF foci mainly oriented towards the apical side of the basal cell layer. Insets show magnified and channel separated views of rectangular selections

To ascertain the significance of our findings in a human tissue context, we conducted immunostainings on 5 µm paraffin sections of cervix biopsies taken from individuals ranging in age from 25 to 63 years, all without diagnosed cervix pathology. While both lamin B1 and lamin A/C were present in the considered samples irrespective of the donor age, many (min. 2 out of 4 sections in 6/10 samples) displayed a conspicuous drop in the lamin B1/lamin A/C intensity ratio, specifically in the basal layer of the stratified epithelium (Fig. 6a). These cells still displayed nuclear lamin B1 signal, albeit of a more diffuse nature. To test whether the reduction in lamin B1 would sensitize the cells to nuclear rupture, we stained a second set of slices for the chromatin binding protein BAF, known to recognize cytoplasm-exposed chromatin. This revealed a prominent focal staining at the nuclear envelope of cells in the basal layer, predominantly at the apical side (min. 2/4 sections of 10/13 samples) (Fig. 6b). From this, we conclude that the basal layer of the cervix epithelium is vulnerable to a selective reduction in lamin B1 and the consequent damage to the nuclear envelope.

## Discussion

In this work, we found that the loss of *LMNB1* sensitizes HeLa and HaCaT cells to HP-PsV infection. Using quantitative live cell imaging of the EGFP reporter signal, we showed that LMNB1ko cells have a faster infection kinetics and higher infection rate, and that individual infected cells accumulate stronger EGFP signals. When examining the potential cause, we found that LMNB1ko cells display increased nuclear perviousness, due to the occurrence of NER and an extended mitotic window.

NER events are not unique to LMNB1ko cells. In fact, the phenomenon was first comprehensively described in laminopathy patient fibroblasts with different mutations in the *LMNA* gene [13] and later also confirmed in cells that completely lack A-type [44] or B-type lamins [45]. However, as we now find, LMNB1ko cells appear to be notoriously sensitive to these events and are also more prone to re-rupturing than the other lamin knockouts (LMNAko, LMNB2ko). This may be due to the specific nuclear defects that are induced by the loss of lamin B1, namely the blebs, which represent weak spots that might be more difficult to repair. Despite the ample NER in LMNB1ko cells and capability of HP-PsV units to enter during such events, we assume that their ephemeral nature makes their contribution to the increased HP-PsV load subordinate to the extension of the mitotic window. The latter may be due to the fact that lamin B1 contributes to mitotic spindle assembly, which is crucial for proper chromosome segregation [46].

While we observed a significant increase in the number of EGFP-positive cells (*i.e.,* infection rate) in HP-PsV infected LMNB1ko cells, and in the maximum EGFP intensity at single cell level, no significant increase in the number of EGFP transcripts was observed in EGFP-positive cells. This could imply that the turnover of transcripts is not defining for the protein load and/or that the translational status can change. However, it could also suggest that the vDNA is less (efficiently) transcribed. This may be due to a stochastic dysregulation of cell homeostasis, as provoked by NER [13], but may also be caused by directed cellular adaptations to the loss of lamin B1. For example, we found that LMNB1ko cells display a higher overall repressed chromatin state, especially in blebs. This is likely due to the accumulation of LADs, genome regions with enrichment of heterochromatin marks (H3K9me3, H3K27me3) and reduced accessibility, as also observed upon loss of lamin A acetylation [57]. Other groups have reported varying effects on the abundance of euchromatin and facultative heterochromatin, but this may be related to differences in experimental models [47–49]. Irrespective of the impact of *LMNB1* knockout on the H3K9me2,3/H3K9ac ratio, it is not known whether the global epigenetic state of the host chromatin also defines the transcription of a rogue episome. More likely, the reduction of PML bodies in LMNB1ko cells is responsible for the lower vDNA transcription. One possible explanation for the reduced PML grafting in LMNB1ko cells could be the translocation to the cytoplasm and gradual dissolution of PML bodies during NER, as observed in laminopathy patient fibroblasts [13]. Another possibility is that the prolonged mitotic window in *LMNB1ko* cells promotes PML body dynamics (when their chromatin association is lost) and delays their recruitment to the nucleus [50].

The limited transcriptional impact in LMNB1ko cells made us examine whether protein turnover was dysregulated. This revealed a significant reduction of autophagic capacity. At the same time, cGAS persistently localized to putative rupture sites (*i.e.*, nuclear blebs) in lamin B1 depleted cells. While cGAS is required for dsDNA-induced autophagy, autophagy itself contributes to its degradation to avoid overactivation [51, 52]. A sustained detection of self-DNA by cGAS and failure to lower this elicitor by autophagy in LMNB1ko cells may eventually lead to a desensitization of this pathway to safeguard cell homeostasis and genome integrity (by preventing DNA damage) [53]. In contrast with another study, we did find a TBK1-mediated response to HP-PsV infection [55], albeit modest, which may be caused by differences in cell type or pseudoviral load. While this canonical (TBK-1-mediated) interferon-response is still maintained upon the detection of HP-PsV vDNA, the shortcut in autophagy signaling via cGAS/STING, which is TBK-1 independent [54], accelerates the buildup of capsid proteins. Therefore, we assume that the desensitization of cGAS dampens autophagic capacity in lamin B1 depleted cells. Together with the increased nuclear access, this could explain the amplified HP-PsV infection rate.

Despite their ample use in relevant HPV infection studies [7, 25, 42], HeLa cells contain multiple copies of integrated HPV18 DNA, which might influence their response to HPV infection [43]. However, we have shown that HaCaT cells respond as hypersensitive to HP-PsV infection and show a similar autophagic defect as HeLa cells upon LMNB1 kd, indicating that lamin B1 curtails early HPV infection in a cell-independent manner. Furthermore, we unexpectedly found that lamin B1 levels are specifically lowered in the basal layer of the cervix epithelium and that this is associated with focal BAF accrual at the NE, suggesting that this pathway has in vivo significance even in healthy individuals. Since BAF has been shown to restrict cGAS recognition of genomic DNA, its mobilization may represent a safeguard mechanism against aberrant immune activation [56]. However, given its widespread occurrence, it is conceivable that persistent nuclear damage puts a strain on the cells’ buffering capacity. Finally, our findings may have important implications for other DNA viruses that exploit the host cell’s nuclear replication and/or transcription machinery for their amplification. Indeed, while HPV depends on PML for its efficient transcription and may therefore not fully profit from the enhanced nuclear access, other viruses that do not need or even target and dissociate PML NBs for establishing productive infection [38] may thrive even more in such a setting.

### Supplementary Information

Below is the link to the electronic supplementary material.

Supplementary file1 (PDF 7259 KB)

## Data Availability

All results are provided in the manuscript and accompanying supplemental file. Established cell lines are available upon request. Image analysis code is available on GitHub (https://github.com/verschuurenM and https://github.com/DeVosLab/).
