# Supplementary material for: Lamin B1 curtails early human papillomavirus infection by safeguarding nuclear compartmentalization and autophagic capacity
Source: Cell Mol Life Sci. 2024 Mar 14;81(1):141. doi: 10.1007/s00018-024-05194-3 (PMC10940392; doi:10.1007/s00018-024-05194-3)
Supplement: Supplementary file 1 — Supplementary file1 (PDF 7259 KB) [file 18_2024_5194_MOESM1_ESM.pdf]

Supplementary Material

Lamin B1 attrition promotes early human papillomavirus infection through increased nuclear access and blunted autophagic capacity.

Freya Molenberghs[1], Marlies Verschuuren[1], Lauran Vandeweyer[1], Sarah Peeters[1], Johannes J. Bogers[1], Claudina Perez Novo[2], Wim Vanden Berghe[2], Hans De Reu[3], Nathalie Cools[3], Mario Schelhaas[4], Winnok H. De Vos[1*]

[1] Laboratory of Cell Biology and Histology, Department of Veterinary Sciences and Health Sciences, University of Antwerp, Belgium

[2] Laboratory of Protein Chemistry, Proteomics and Epigenetic Signaling (PPES), Department of Biomedical Sciences, University of Antwerp, Belgium

[3] Laboratory of Experimental Hematology, Faculty Medicine and Health Sciences, University of Antwerp, Belgium

[4] Institute of Cellular Virology, University of Münster, Germany

Contact information:

*Corresponding author:

University of Antwerp

Laboratory of Cell Biology and Histology

Universiteitsplein 1, 2610 Antwerp, Belgium

e-mail: winnok.devos@uantwerpen.be

Supplementary Table 1 Overview p values of all statistical comparisons shown in the figures.

**Fig. S1c**	**x**	**y**	**p value**	**Sign.**		**x**	**y**	**p value**	**Sign.**
Nuclear circ.	CTRLko	LMNAko	<1^{e}-10	***	Mean lamin A/C int.	CTRLko	LMNAko	<1^{e}-10	***
	CTRLko	LMNB1ko	<1^{e}-10	***		CTRLko	LMNB1ko	<1^{e}-10	***
	CTRLko	LMNB2ko	<1^{e}-10	***		CTRLko	LMNB2ko	<1^{e}-10	***
	LMNAko	LMNB1ko	<1^{e}-10	***		LMNAko	LMNB1ko	<1^{e}-10	***
	LMNAko	LMNB2ko	<1^{e}-10	***		LMNAko	LMNB2ko	<1^{e}-10	***
	LMNB1ko	LMNB2ko	<1^{e}-10	***		LMNB1ko	LMNB2ko	<1^{e}-10	***
**Fig. 1a**	**x**	**y**	**p value**	**Sign.**		**x**	**y**	**p value**	**Sign.**
Infection rate	CTRLkd	LMNAkd	0.99913	n.s.	Mean EGFP int.	CTRLkd	LMNAkd	0.98887	n.s.
	CTRLkd	LMNB1kd	<0.001	***		CTRLkd	LMNB1kd	<0.001	***
	CTRLkd	LMNB2kd	0.99801	n.s.		CTRLkd	LMNB2kd	0.99070	n.s.
Infection rate	CTRLko	LMNAko	0.166	n.s.	Mean EGFP int.	CTRLko	LMNAko	0.1182	n.s.
	CTRLko	LMNB1ko	<0.001	***		CTRLko	LMNB1ko	<0.001	***
	CTRLko	LMNB2ko	0.989	n.s.		CTRLko	LMNB2ko	1.000	n.s.
**Fig. 1c**	**x**	**y**	**p value**	**Sign.**		**x**	**y**	**p value**	**Sign.**
Infection rate	CTRLkd	LMNB1ko	0.00645	**	EGFP intensity	CTRLko	LMNB1ko	0.00322	**
**Fig. 2a**	**x**	**y**	**p value**	**Sign.**		**x**	**y**	**p value**	**Sign.**
Doubling time kd	CTRLkd	LMNAkd	0.94	n.s.	Doubling time ko	CTRLko	LMNAko	0.88	n.s.
	CTRLkd	LMNB1kd	0.94	n.s.		CTRLko	LMNB1ko	0.88	n.s.
	CTRLkd	LMNB2kd	0.94	n.s.		CTRLko	LMNB2ko	0.88	n.s.
**Fig. 2c**	**x**	**y**	**p value**	**Sign.**	**Fig. 2d**	**x**	**y**	**p value**	**Sign.**
Mitotic window	CTRLko	LMNB1ko	9.271^{e}-07	***	EdU spots	CTRLko	LMNB1ko	0.04677	*
**Fig. 2f**	**x**	**y**	**p value**	**Sign.**		**x**	**y**	**p value**	**Sign.**
Fraction cells with ruptures	CTRLko	LMNAko	0.193	n.s.	Rate rerupture	CTRLko	LMNAko	0.921	n.s.
	CTRLko	LMNB1ko	<0.001	***		CTRLko	LMNB1ko	<1^{e}-05	***
	CTRLko	LMNB2ko	1.000	n.s.		CTRLko	LMNB2ko	1.000	n.s.

**Fig. 2f**	**x**	**y**	**p value**	**Sign.**					
EdU<br>spots	CTRLko	LMNB1ko	0.002381	**					
**Fig. S3**	**x**	**y**	**p value**	**Sign.**		**x**	**y**	**p value**	**Sign.**
+HPV PsV	CTRLko	LMNB1ko	0.688	n.s.	EGFP+	CTRLko	LMNB1ko	0.454	n.s.
**Fig. 3a**	**x**	**y**	**p value**	**Sign.**		**x**	**y**	**p value**	**Sign.**
-HPV/	CTRLko	LMNB1ko	0.00479	**	EGFP-	CTRLko	LMNB1ko	0.234	n.s.
+HPV PsV	- HPV	+HPV	0.4271	n.s.	/EGFP+	EGFP-	EGFP+	0.645	n.s.
**Fig. 3c**	**x**	**y**	**p value**	**Sign.**		**x**	**y**	**p value**	**Sign.**
-HPV/	CTRLko	LMNB1ko	<2^{e}-16	***	EGFP-	CTRLko	LMNB1ko	2.6^{e}-12	***
+HPV PsV	- HPV	+HPV	0.15	n.s.	/EGFP+	EGFP-	EGFP+	2^{e}-16	***
**Fig. 3d**	**x**	**y**	**p value**	**Sign.**		**x**	**y**	**p value**	**Sign.**
24h	CTRLko<br>CTRLkd	CTRLko<br>PMLkd	1.000	n.s.	48h	CTRLko<br>CTRLkd	CTRLko<br>PMLkd	0.31976	n.s.
	CTRLko<br>CTRLkd	LMNB1ko<br>CTRLkd	0.01240	*		CTRLko<br>CTRLkd	LMNB1ko<br>CTRLkd	<0.001	***
	CTRLko<br>CTRLkd	LMNB1ko<br>PMLkd	0.99638	n.s.		CTRLko<br>CTRLkd	LMNB1ko<br>PMLkd	0.94222	n.s.
	CTRLko<br>PMLkd	LMNB1ko<br>CTRLkd	0.00666	**		CTRLko<br>PMLkd	LMNB1ko<br>CTRLkd	<0.001	***
	CTRLko<br>PMLkd	LMNB1ko<br>PMLkd	0.98656	n.s.		CTRLko<br>PMLkd	LMNB1ko<br>PMLkd	0.01488	*
	LMNB1ko<br>CTRLkd	LMNB1ko<br>PMLkd	0.11160	n.s.		LMNB1ko<br>CTRLkd	LMNB1ko<br>PMLkd	<0.001	***
**Fig. 4a**	**x**	**y**	**p value**	**Sign.**					
Prot. degr.	DMSO	MG132	<2^{e}-16	***					
**Fig. 4b**	**x**	**y**	**p value**	**Sign.**		**x**	**y**	**p value**	**Sign.**
DMSO	CTRLko<br>HPV PsV-	CTRLko<br>HPV PsV+	0.5669	n.s.	RM/CQ	CTRLko<br>HPV PsV-	CTRLko<br>HPV PsV+	0.869	n.s.
	LMNB1ko<br>HPV PsV-	LMNB1ko<br>HPV PsV+	0.0147	*		LMNB1ko<br>HPV PsV-	LMNB1ko<br>HPV PsV+	0.960	n.s.

	CTRLko<br>HPV-	LMNB1ko<br>HPV PsV-	0.9982	n.s.		CTRLko<br>HPV-	LMNB1ko<br>HPV PsV-	<1e-05	***
	CTRLko<br>HPV PsV+	LMNB1ko<br>HPV PsV+	0.4210	n.s.		CTRLko<br>HPV PsV+	LMNB1ko<br>HPV PsV+	<1e-05	***
**Fig. S5**	**x**	**y**	**p value**	**Sign.**		**x**	**y**	**p value**	**Sign.**
DMSO	CTRLko	LMNB1ko	0.000653	***	RM/CQ	CTRLko	LMNB1ko	0.00628	**
**Fig. 5c**	**x**	**y**	**p value**	**Sign.**		**x**	**y**	**p value**	**Sign.**
-HPV/<br>+HPV PsV	CTRLko	LMNB1ko	0.516	n.s.	EGFP-<br>/EGFP+	CTRLko	LMNB1ko	0.847	n.s.
	-HPV	+HPV	0.124	n.s.		EGFP-	EGFP+	<2e-16	***
**Fig. 5d**	**x**	**y**	**p value**	**Sign.**		**x**	**y**	**p value**	**Sign.**
-HPV/<br>+HPV PsV	CTRLko	LMNB1ko	0.0238	*	EGFP-<br>/EGFP+	CTRLko	LMNB1ko	0.0231	*
	-HPV	+HPV	0.00814	**		EGFP-	EGFP+	<2e-16	***

Supplementary Figures

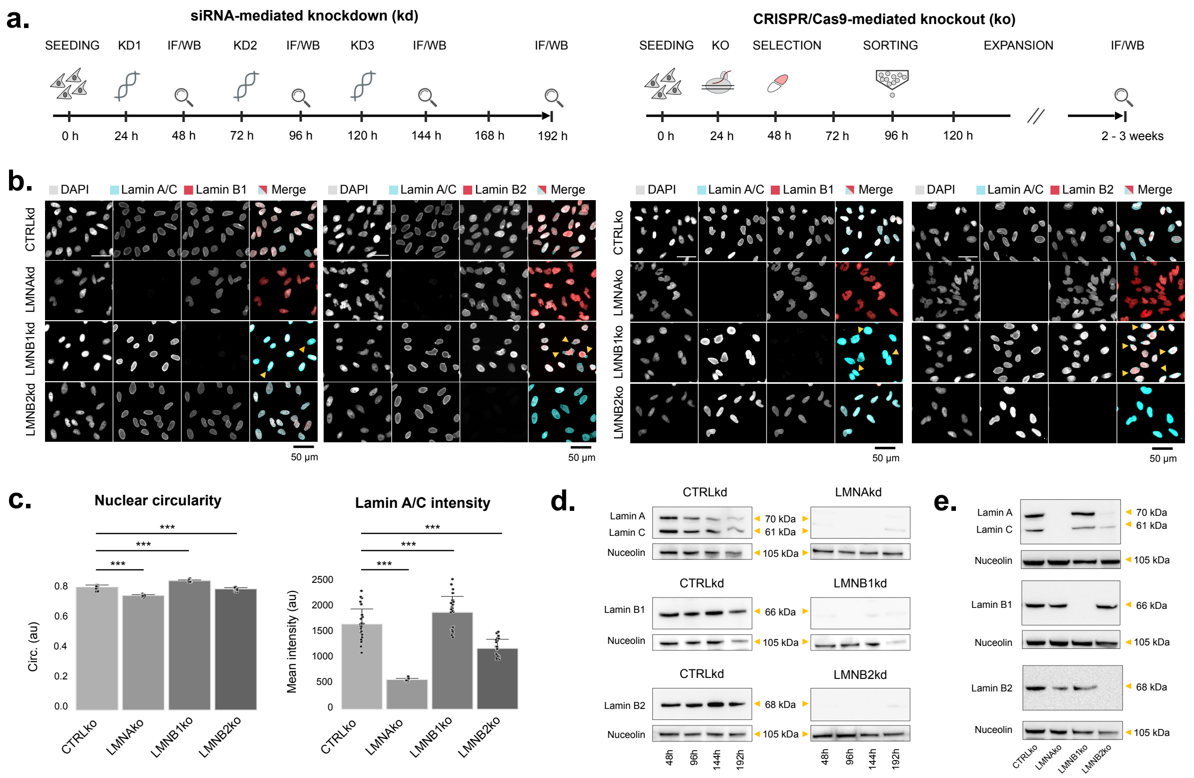

Supplementary Figure S1. Loss of individual lamins provokes distinct morphological changes. **a)** Workflow for establishment of lamin-depleted cells. Repetitive siRNA-mediated genome silencing was used to establish a sustained knockdown (kd). HeLa cells were seeded at time point 0 h and three subsequent knockdowns were performed on 24 h, 72 h and 120 h. At different timepoints after cell seeding (*i.e.*, 48 h, 96 h, 144 h and 192 h), readouts were obtained by immunofluorescent staining (IF) and western blot (WB). CRISPR/Cas9 genome editing was used to obtain stable knockout (ko) cells. HeLa cells were seeded and CRISPR/Cas9 constructs were transfected 24 h later. Puromycin was used to enrich transfected cells prior to single cell selection with FACS. Clonal expansion was followed by quantification of these different colonies by IF and WB; **b)** IF reveals the absence of the protein of the respective target genes and reveals significant morphological nuclear changes (insets). While CTRL cells mainly display oval-shaped nuclei, *LMNA* depleted cells show dysmorphic nuclei with local loss of B-type lamins. Depletion of *LMNB1* provokes ample nuclear blebs and yields an increased lamin

A/C signal. Depletion of *LMNB2* does not result in overt changes; **c)** Nuclear circularity and lamin A/C levels of lamin-depleted cells differ significantly from CTRL cells ($n_{bio} = 3$, $n_{tech} = 5$, $p < 0.05$, linear mixed effects model); **d)** WB confirms the virtual absence of the target proteins in sustained kd as well as in **e)** stable ko cells.

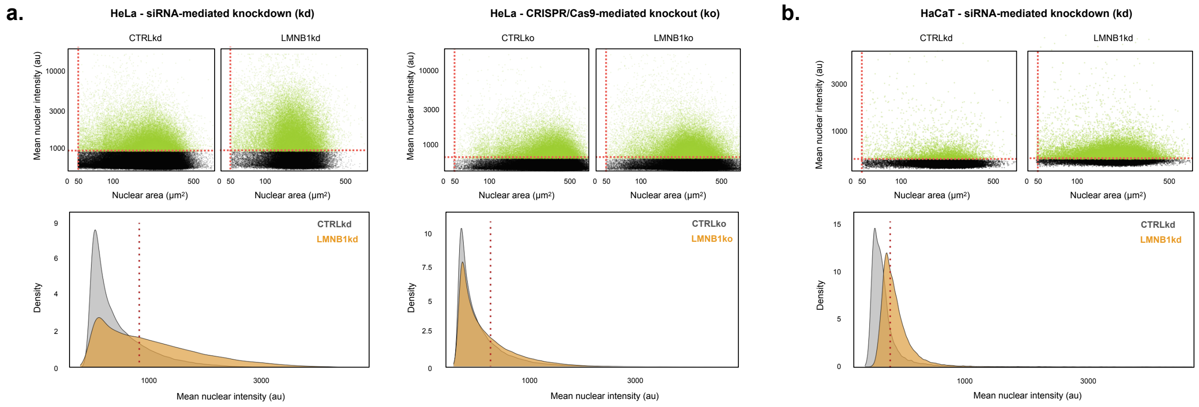

Supplementary Figure S2. Scatterplots and corresponding density plots illustrating the distribution of EGFP intensity as a function of the nuclear area of HP-PsV infected HeLa **(a)** and HaCaT **(b)** cells with and without depletion of lamin B1. The red dotted lines indicate fixed cutoffs for cell detection (min. area) or positively infected cells (min. EGFP intensity) (n_{bio} = 2).

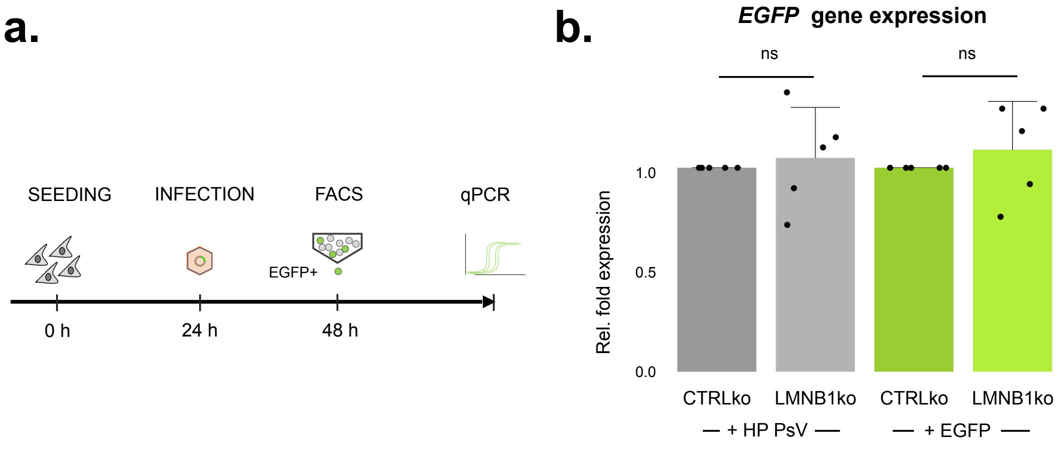

Supplementary Figure S3. HP-PsV-infected *LMNB1* depleted cells do not show higher EGFP gene expression levels. **a)** Workflow to determine the number of EGFP transcripts. CTRLko and LMNB1ko cells were infected with HP-PsV for 24 h before cells were sorted with FACS based on their EGFP-signal. Stringent selection criteria were used to sort true EGFP-positive (EGFP+) and -negative (EGFP-) cells. RNA was collected from the selected cell populations as well as from pooled, non-sorted HP-PsV infected CTRLko and LMNB1ko cells and used for qPCR; **b)** The number of EGFP transcripts in LMNB1ko cells (expressed as fold change with respect to the CTRL and normalized to the reference genes, 2^ddCt) is not significantly increased, not in pooled conditions, nor in flow-sorted EGFP-positive cells (n = 5, one sample T-test, with Shapiro-Wilk normality test).

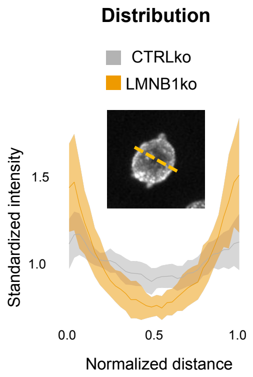

Supplementary Figure S4. LMNB1ko cells show a higher accumulation of perinuclear methylated chromatin marker. The intensity of H3K9me23 is measured along the distance-normalized cross section of the nucleus and is standardized by the mean intensity per nucleus (n=20 cells).

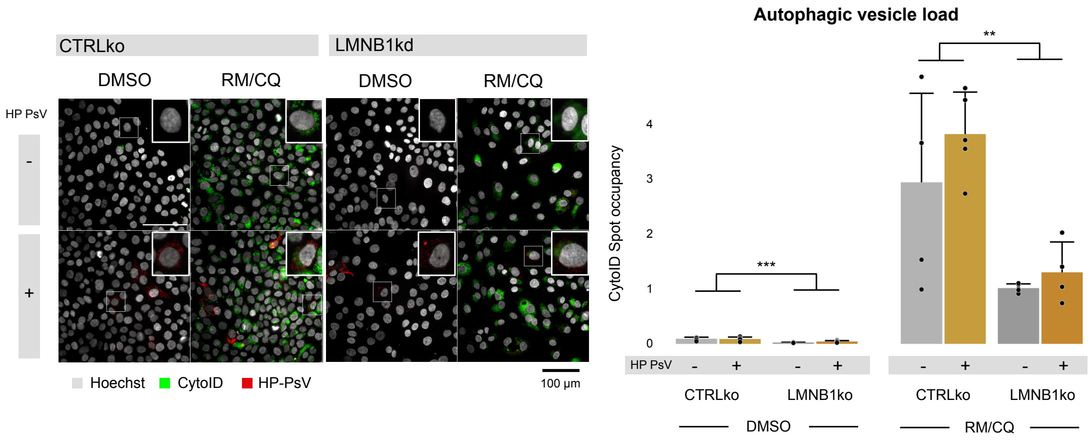

Supplementary Figure S5. Autophagic capacity is blunted in *LMNB1* depleted HaCaT cells. Fluorescent images of cells stained with CytoID show strong enrichment of autophagic vacuoles after treatment with a combination of Rapamycin (RM) (*i.e.,* inducer of autophagy) and Chloroquine (CQ) (*i.e.,* inhibitor of lysosomal degradation) in CTRLko cells, but much less prominent in LMNB1ko cells. This significant difference in autophagic capacity is also confirmed in the quantification of the cellular CytoID spot occupancy (n_{bio} = 3, n_{tech} = 5, two-way Anova with Tukey post hoc).
